# Role of Reactive Silica Addition in Enhancing Geopolymerization Efficiency and Strength Development of Calcined Granite Waste

**DOI:** 10.3390/ma19050886

**Published:** 2026-02-27

**Authors:** Yang Liu, Cao Bi, Yuting Gao, Frederick Ntim Gyakari, Xiaoxiong Zha

**Affiliations:** 1 Shenzhen Construction Engineering Group Co., Ltd., Shenzhen 518055, China; 2 School of Intelligent Civil and Ocean Engineering, Harbin Institute of Technology, Shenzhen 518055, China; 3 Zhejiang Jinggong Steel Structure Group Co., Ltd., Shaoxin 312030, China; 4 Guangdong Provincial Key Laboratory of Intelligent and Resilient Structures for Civil Engineering, Shenzhen 518055, China

**Keywords:** geopolymerization, alkali activation, reactive silica, reactivity, mechanical performance

## Abstract

This study examined the geopolymerization behavior of granite waste powder and reactive silica powder (GWS), utilizing granite waste powder as a sustainable precursor material, to develop an environmentally friendly substitute for Ordinary Portland cement. To obtain this objective, a total of three different mixes of calcined granite waste with reactive silica (1:1, 3:2, 7:3) were cast to evaluate the aim of this study. Due to low inherent reactivity of granite waste powder, the alkali activation was achieved using a combined solution of alkali activators consisting of 8 mol/L concentration of NaOH and Na_2_SiO_3_ solution at mass ratio of 1:1.2 prepared 24 h in advance to ensure complete dissolution and stabilization prior to pouring it into the GWS paste. The finest particle size distribution for optimal reactivity performance was achieved by choosing lowest median particles size from 4.0 μm–4.2 μm among all mixtures. ICP-MS analysis of granite waste and reactive silica showed the presence of silica (0.11% and 0.26% respectively) and calcium (49.61% and 38.92% respectively) content adequate for effective geopolymerization of the paste. The elemental composition, new phase formation and microstructural analysis were examined using X-ray diffraction (XRD), Fourier-transform infrared spectroscopy (FTIR) techniques and Scanning Electron Microscopy (SEM) analysis. XRD analysis revealed that all GWS mixes were predominantly amorphous, with crystalline quartz, feldspar and minor α-cristobalite peaks diminishing from GWS50 to GWS70 confirming increased reactivity due to enormous reactive silica content. FTIR spectra of GWS mixes displayed characteristics of O-H (3375 cm^−1^), H-O-H (1645 cm^−1^), and Si-O-T (982–1000 cm^−1^) bands, with the main Si-O-T peak shifting to higher wavenumbers from GWS50 to GWS70 due to increased GW content, indicating reduced geopolymerization effect in GWS50. SEM analysis revealed that among all mixes, GWS70 exhibited the most ideal dense matrix with increasing content of granite waste along with strong N-A-S-H gel formation. Compressive strength at 28 days increased from 11.2 MPa for GWS50 to 14.2 MPa for GWS60 and 13.8 MPa for GWS70, demonstrating that higher reactive silica powder content significantly enhanced the mechanical performance of the alkali-activated paste. These findings demonstrated that alkali-activated geopolymers of GSW offer a viable alternative to Ordinary Portland cement with optimized mixes by valorizing industrial waste and reducing reliance on high-carbon cement production.

## 1. Introduction

The production of ordinary Portland cement (OPC) remains one of the most carbon-intensive industrial processes, contributing approximately 8% of global CO_2_ emissions, with worldwide annual production projected to reach 5.9 billion tons and emit around 4.8 billion tons of CO_2_ by 2030 [1,2]. This environmental concern triggered an urgent attention for decarbonization of the construction sector and accelerated the adoption of supplementary cementitious materials (SCMs) that can partially replace OPC while enhancing the mechanical and durability performance of concrete [3]. Well-established SCMs, including fly ash [4], metakaolin [5], silica fume [6], ground-granulated blast-furnace slag [7], rice husk ash [8], sugarcane bagasse ash [9], limestone powder [10], walnut shell ash [11], clay-waste [12] and waste paper [13], participate in pozzolanic reactions whereby amorphous silica reacts with calcium hydroxide during cement hydration to produce additional calcium silicate hydrate (C-S-H), thereby refining pore structure, increasing long-term strength, and improving resistance to chemical attack [14,15]. Among these, silica fume stands out for its extremely high reactivity and ability to dramatically densify the microstructure and mitigate durability issues such as sulphate attack and chloride penetration [16].

Similarly, locally abundant natural and industrial by-products are increasingly being explored to further reduce the environmental footprint of cementitious materials. Granite waste (GW), a fine-grained, highly weathered granitic soil rich in silica and alumina, is extracted in massive quantities worldwide and is particularly prevalent in South China and Hong Kong [17], where it covers more than 30% of the land area with thicknesses ranging from 0 to 30 m [18,19]. Chemically, GW is dominated by quartz and clay minerals, with SiO_2_ typically exceeding 68% of the oxide composition, Al_2_O_3_ as the second most abundant oxide, and minor amounts of Na_2_O, K_2_O, CaO, and Fe_2_O_3_ (2–5%) [20]. Because of its abundance, low cost, and inherent pozzolanic potential when suitably activated or combined with other reactive phases, GW has been investigated both as a partial replacement for natural fine aggregates, often improving workability, ductility, and cost efficiency. Also, it performed as an active binder component capable of contributing to long-term strength gain through secondary hydration reactions [21,22,23]. Recent research has also demonstrated that highly reactive amorphous silica sources, such as silica fume, can effectively replace conventional alkaline activators (e.g., sodium silicate) in geopolymeric systems while simultaneously enhancing mechanical properties and promoting industrial waste valorization [24,25,26].

Despite these individual successes, significant knowledge gaps persist regarding the synergistic interaction between naturally weathered materials like GW and highly reactive silica particles in blended cementitious systems. In particular, the precise chemical reaction pathways, the extent of silica-driven pozzolanic activity, the formation of secondary C-S-H gel, the evolution of microstructure over extended curing periods, and the resulting impact on macroscopic mechanical and durability properties still require more exploration for silica fume. Without a comprehensive mechanistic understanding, the full potential of combining locally available granite waste with reactive silica to produce high-performance, low-carbon cementitious composites cannot be confidently realized or standardized for practical engineering applications.

Therefore, the present study aims to systematically explore the underlying reaction mechanisms in cementitious systems incorporating granite waste and reactive silica powder (GSP), with particular emphasis on the role of silica-driven pozzolanic activity in microstructural densification, phase assemblage development, and long-term performance enhancement. Through detailed characterization of hydration products, pore structure refinement, and mechanical property evolution, this work seeks to establish a robust scientific foundation for the design of sustainable, regionally sourced building materials that substantially reduce reliance on ordinary Portland cement while delivering comparable or superior strength and durability characteristics [13]. By bridging the gap between abundant local mineral resources and high-reactivity industrial by-products, the research ultimately contributes to the development of environmentally responsible construction materials capable of meeting both performance requirements and global CO_2_ reduction goals.

## 2. Materials and Methods

### 2.1. Materials Preparation

**Figure 1:**
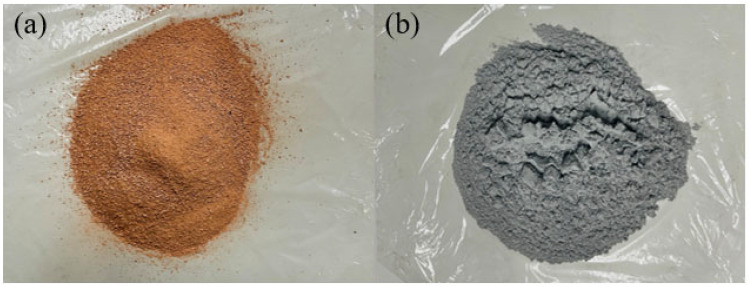
(**a**) Granite waste and (**b**) reactive silica.

The granite waste mineralogically dominated by quartz and feldspar possessing a chemical profile in high in silica (SiO_2_) and alumina (Al_2_O_3_) with notable amounts of CaO, K_2_O, and Fe_2_O_3_ was sourced from Shenzhen Metro construction site and was taken through various steps during preparation as shown in Figure 1. These stages included drying, crushing, and sieving. This is to ensure the homogeneity of the particle size distribution that is suitable for subsequent use in cementitious composites. The raw GW was oven-dried at 105°C for 24 h to remove moisture, as the presence of water can influence the reaction rates. The dried GW was lightly pounded and then sieved out. Particle sizes predominantly around 60 µm and below were selected for the experiment. The reactive silica powder utilized was commercially available silica fume. The silica fume contained more than 82% SiO_2_ and particle size ranging from 0.075 µm to 0.225 µm. The RSP was sourced from Shanghai Shanying Environmental Protection Technology Co., Ltd., Shanghai, China and was readily available for use in the lab. They were then put together according to the various predetermined mix ratios and calcinated for 2 h at 700 °C at a rate of 30°C.

Although the reactive silica powder has excellent pozzolanic potential, calcined granite waste powder has low inherent reactivity due to its crystalline structure due to which it exhibits limited dissolution and poor reactivity performance in geopolymers. To obtain the highest reactivity of GSW, the alkali activation was highly essential in the binder mixes. Alkali-activated pastes were prepared from calcined granite waste and reactive silica powder at GW: RSP proportions of 1:1, 3:2, and 7:3 as illustrated in Table 1. Cubic molds 30 × 30 × 30 mm were used. Calcined granite waste and reactive silica were mixed according to the ratios with respect to the total binder mass. The blend was dry mixed and homogenized in a planetary mixer at low speed for 3 min to achieve uniform particle distribution, essential for uniform pozzolanic reactivity, and optimal hydration. The alkaline activator solution NaOH/Na_2_SiO3 ratio of 1:1.2 was prepared 24 h ahead and cooled to room temperature. The pre-mixed binder was then gradually added to the activator while mixing at low speed for 3 min, then at high speed for another 3 min to form a uniform workable paste. The paste was poured into 30 × 30 × 30 mm steel cube molds and compacted on a vibrating table for 30–45 s to remove entrapped air. The surface was leveled, and the molds were covered with plastic film to prevent moisture loss. The specimens underwent initial curing at room temperature for 48 h, after which they were demolded. They were then placed in an oven for curing at 75°C for 1 h (heat curing), and then room temperature curing until testing age for compressive strength at (3, 7, and 28 days). This ensured consistent preparation across all the GW-RSP alkali-activated mixes [27].

### 2.2. Particles Size Distribution

**Figure 2:**
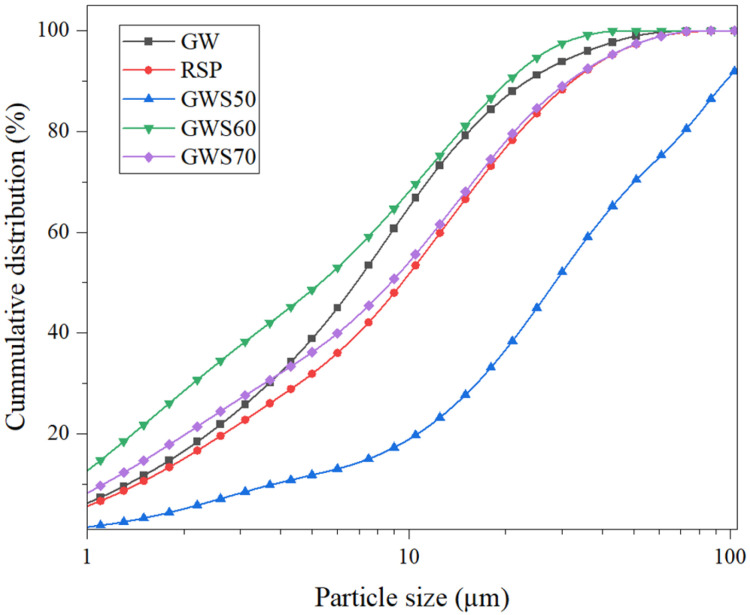
Particle size distribution of granite waste (GW), reactive silica powder (RSP) and GWS mixes.

Particle size distribution for materials’ granite waste and reactive silica was selected with the ratios (GW: RSP 1:1, 3:2, 7:3) as different mixes were illustrated in Figure 2. Granite waste showed the coarsest particles (60 µm) approximately with prominent tails that limited the reactivity, while RSP more finely facilitated the pozzolanic reactivity in the mix. As the proportion of GW increased from GWS50 to GWS70, the cumulative particle size distribution curves shifted to the right, providing clear evidence of the systematic coarsening of the mix, while GWS50 exhibited the finest overall distribution and GWS70 the courser among the three mixes. Higher GW content increased particle size, reducing packing efficiency due to the coarser gradation, larger voids, and lower bulk density. This raised alkali demand to maintain workability and maintain the reactivity of the geopolymer mix [28,29]. Thus, blending of GW and RSP particles for all three mixes showed improved particles size in comparison with granite waste making GWS50 an ideal mix.

### 2.3. Element Composition of GW-RSP Reactivity

**Figure 3:**
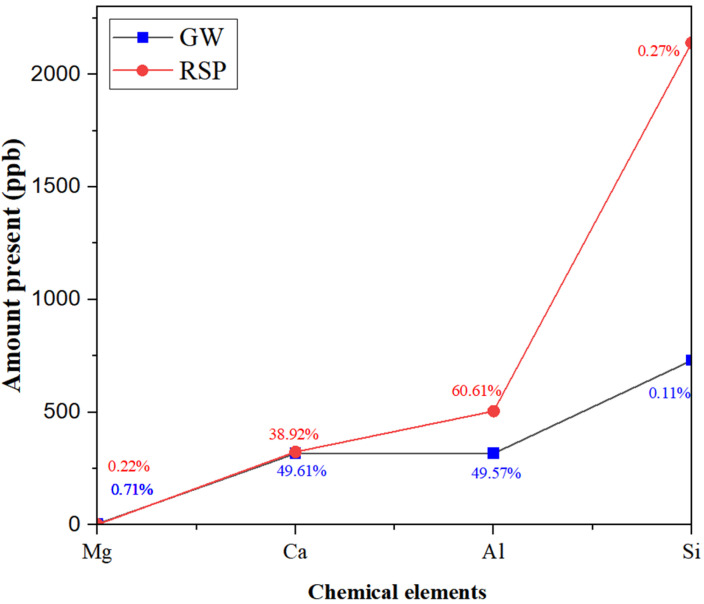
Phase dissolution of granite waste and reactive silica powder.

ICP-MS testing was performed using Inductively Coupled Plasma Mass Spectrometry (ICP-MS) (iCAP RQ manufactured by Thermofisher Scientific Co Ltd., Bremen, Germany). The test showed that calcined granite waste (GW) is predominantly silicon-based, accompanied by a notable aluminum content and a smaller calcium fraction, while reactive silica powder (RSP) is composed almost exclusively of silicon, with only minimal traces of aluminum and calcium, as shown in Figure 3. The silicon level in RSP far exceeds that of GW, and calcium and aluminum are distinctly more abundant in GW [30]. The ICP-MS test on reactive silica derived from industrial by-products confirmed exceptionally high silicon with low amounts of calcium and aluminum, which is consistent with the RSP profile [31]. Comparative ICP-MS analyses of blended precursors for alkali-activated binders showed that pairing a silicon-rich reactive component with a calcium- and aluminum-containing calcined waste creates balanced elemental proportions conducive to effective geopolymerization and strength gain [32]. Thus, the complementary chemical characteristics of GW and RSP, as validated by ICP-MS data, provide a solid scientific foundation for the selected GW-RSP blending ratios in the present alkali-activated pastes.

## 3. Material Characterization

GW-RSP binders were characterized by various techniques to assess phase composition, chemical bonding, and structural formation within the different mixes of GWS. For XRD and FTIR analysis, 30 × 30 × 30 mm alkali-activated GWS cubes were first crushed into small fragments to halt further reactions, followed by solvent exchange with isopropanol and gentle drying to preserve the phase composition. The dried samples were then manually ground to fine power to ensure the particles’ homogeneity. A small portion of the resulting powder was placed in XRD holder to achieve a flat surface and random crystal orientation for accurate diffraction analysis. XRD patterns were recorded using a Cu-kα diffractometer (manufactured by Bruker AXS GmbH, Karlsruhe, Germany) (40 kV, 40 mA) to identify crystalline phases and determine lattice parameters of the paste. Concurrently, the same methodology was applied for FTIR results as homogeneous mixture was pressed into a translucent pellet under high pressure for transmission-mode FTIR spectroscopy to obtain clear vibrational spectra characteristic of the geopolymer bonds. FTIR Spectrometer (Spectrum 100, Perkin Elmer Inc, Shelton, WA, USA) was used to identify characteristic vibrational modes associated with functional groups, determining the chemical bonds, molecular structures, and intermolecular interactions [33]. A scanning electron microscopy (SEM) test was performed on all the different ratios of mixes using Zeiss Gemini 300 X-MAXN manufactured by Carl Zeiss Microscopy GmbH, Jena, Germany.

### Mechanical Characterization

The alkali-activated GW-RSP binder’s compressive strength tests were conducted on 30 × 30 × 30 mm cubes of paste GW: RSP proportions of 50:50, 60:40, and 70:30 under a constant 3 KN load according to the standard ASTM C109/C109M-21 using CRIMS load testing machine manufactured by SINOTEST Equipment Co., Ltd. Changchun, China [34]. The total number of specimens were 27 and 9 specimens for each mix, tested at a curing age of 3 days, 7 days and 28 days. Mechanical strength was calculated as

σ = P/A(1)

Up to 28 days of curing. Results at 3, 7, and 28 days showed distinct strength development for each mix, revealing the influence of mix proportions on the material mechanisms and structural integrity.

## 4. Results and Discussion

### 4.1. XRD

**Figure 4:**
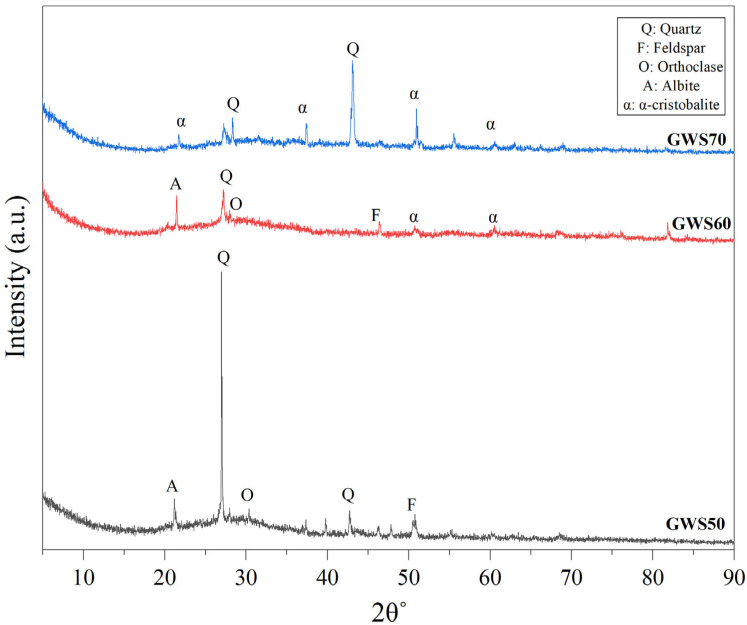
X-ray diffraction (XRD) analysis of granite waste with reactive silica in different ratios.

The XRD pattern of alkali-activated granite waste with reactive silica (GWS) in different ratios revealed a clear evolution in composition from silica-rich to granite-rich formulations, as illustrated in Figure 4. The most intense peaks in all three samples were quartz, showing strong reflection at 20.9° and 26.6°, but their intensities decreased from GWS50 to GWS70 because reactive silica was the primary Quartz/cristobalite source, while granite waste itself contributed only 25–35% Quartz and instead supplied highly reactive feldspar [35]. The quartz in GWS50 remained inert during alkali activation due to its thermodynamic stability, taking part only in dissolution by releasing minor soluble silica, which acted as microaggregates. These microaggregates enhanced dimensional stability and abrasion resistance in GWS60 but slightly enhanced the porosity in GWS70 (obvious from big peaks) due to not fully encapsulating the gel as seen in previous work [36]. On the other hand, the decrease in silica quantity in GWS60 and GWS70 revealed that feldspar peaks, orthoclase, and albite peaks between 27 and 28° became more prominent, reflecting the natural abundance of albite microcline in granite waste [37]. Unlike highly resistant quartz and cristobalite, these aluminosilicate feldspars were more susceptible to alkaline attack as they underwent rapid amorphization and released a substantial amount of Si and Al ions, which served as the primary source of geopolymer network gel formation. This N-A-S-H gel formation is evidenced by the peaks at 25–35° in GWS60 and GWS70, indicating a progressive intensification of the amorphous hump reaching its prominent and well-defined stable state, especially in GWS60.

The α-cristobalite peaks showed reflections between 20 and 22°, with the strongest peaks in GWS70, confirming that silica powder significantly consisted of a high-temperature crystalline fraction that dissolved only more than quartz, supplying more soluble silica in GWS60 and GWS70, proving beneficial in a low alkaline system [38]. NaOH and Na_2_SiO_3_ were the main reasons for faster dissolution of silica, as they preferably attacked the feldspar and surfaces of quartz and provided additional silicates that accelerated the polycondensation, resulting in a strong gel formation represented by sharp peaks in GWS60 and GWS70, as aligned with previous studies [39]. The rearrangement of quartz peaks at 26° with multiple feldspar peaks at 27–30° in GWS60 and GWS70 reflected that the sample mix became denser, consisting mostly of aluminosilicate feldspar, but an excessive amount of feldspar reduced the microstructural bonding networks, resulting in lower mechanical strength (compressive strength results), especially in GWS70. Consequently, minor α-cristobalite peaks became more visible and the broad amorphous hump (25–35°), indicating enhanced geopolymer gel formation (C-(N)-A-S-H/N-A-S-H) driven by increased Al^3+^ release from feldspars and quartz in all sample peaks.

In short, the XRD pattern of all GWS mixes showed compositional changes from GWS50 to GWS70 as quartz and α-cristobalite gradually decreased due to low silica content, while feldspar peaks (albite, orthoclase) became progressively stronger due to high content of granite waste. Overall, a high granite waste proportion improved the degree of polymerization despite the low mechanical stability and weak microstructural bonding in GWS70.

### 4.2. Functional Group Analysis

**Figure 5:**
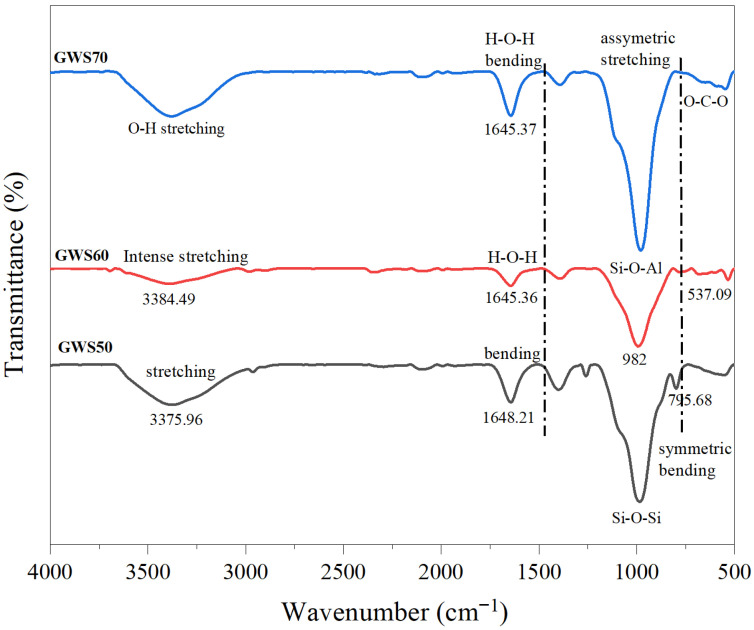
Functional group formations analysis by FTIR.

The FTIR spectra of GWS samples as presented in Figure 5, revealed the evolution of new functional groups formation during geopolymerization, with transmission curves showing progressive shifts and intensity changes, with the increase in granite waste ratio from 50% to 70%. In the GWS50 curve, a stretching band centered at 3376 cm^−1^ indicated weakly hydrogen-bonded hydroxy groups from free water and silanol (Si-OH) surfaces of undissolved silica particles, with lower intensities suggesting limited hydration as compared to GWS60 and GWS70. The same pattern is further followed by a sharp H-O-H blending vibration at 1648 cm^−1^, characteristics of molecular water in pores, and a dominant asymmetric Si-O-Si stretching peak at 982 cm^−1^, signifying highly polymerized silica tetrahedra (Q^4^ environment) from silica powder in GWS50. The minor shoulders at 745 cm^−1^ Si-O-Si symmetric blending indicate incomplete geopolymerization with a silica-dominated, leading to higher porosity in GWS50 [40].

While in the GWS60 curve, the O-H band broadened and shifted slightly to 3384 cm^−1^ with lower intensity, indicating stronger hydrogen bonding from increased Al-OH and Si-OH groups released from partial feldspar dissolution in granite waste. These feldspars further helped in enhancing water retention and early gel formation, and sharpened the H-O-H peak 1645 cm^−1^, pointing to more bound water in forming N-A-S-H gel, while the main band split into a prominent Si-O-Al asymmetric stretch at 982 cm^−1^, and a shoulder at 537 cm^−1^ confirmed the balance Si/Al ratio leading to denser microstructure through polycondensation [41,42]. The GWS70 curve exhibited the most intense and broad O-H stretching at 3384 cm^−1^, reflecting extensive hydrogen bonding networks from abundant Al-OH in dissolved albite, promoting massive hydrogen shells. The H-O-H bending at 1645 cm^−1^ reflected the trapped water in maturing C-A-S-H gel formation, and the Si-O-Al peak at 982 cm^−1^ dominated with lower transmittance, showing a high Al ratio from granite waste content. The peak at 537 cm^−1^ exhibited O-C-O bending formation from calcination of granite waste during material preparation, resulting in the lowest porosity due to highly charged and fine particles of granite waste [38]. At the same time, alkali activators played a significant role in catalyzing all these transformations by elevating pH > 12, hydrolyzing granite waste aluminosilicates (feldspar > quartz) to release Si and Al ions that condensed into Si-O-Al group formations. Furthermore, they helped in shifting Si-O-Si vibrations to lower wavenumbers by forming functional groups such as Si-O and Al-O-Si, which evolved from Q^4^ in GWS50 to Q^3^ and Q^2^ in GWS70 for a 3D tetrahedral network [39]. This is evidenced in GWS60 and GWS70 curves by decreasing Si-O-Si intensity and emerging Si-O-Si shoulders in the curves, minimizing unreacted phases and efflorescence.

In short, increasing granite waste significantly weakened the sharp Si-O-Si peaks while intensifying the O-H and H-O-H bands, reflecting well dissolution and hydration. Overall, higher granite waste content showed the transition from silica-dominated, poorly reacted structure (GWS50) to high geopolymer mix (GWS70) with enhanced and superior binding properties in GWS60 and GWS70.

### 4.3. SEM Analysis

**Figure 6:**
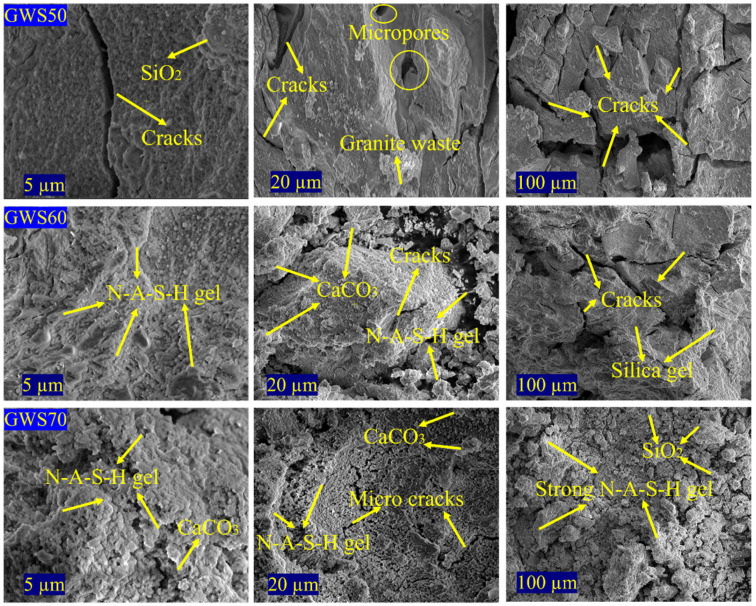
SEM analysis of GWS50, GWS60 and GWS70.

The SEM images of alkali-activated geopolymer samples of GWS in different ratios revealed distinct microstructural evolution with increasing content of granite waste as illustrated in Figure 6. In GWS50, the 5 μm scale showed prominent SiO_2_ particles and extensive cracks, while 20 μm and 100 μm displayed unfilled micropores and larger cracks, and fragmented granite waste aggregates, reflecting incomplete matrix densification and limited gel formation due to excessive reactive silica diluting the aluminosilicate reactivity under alkali activation. GWS60 exhibited N-A-S-H gel, and CaCO_3_ precipitates at 5 μm with some cracks, a denser matrix than GWS50 with persistent cracks at 20 μm, and silica gel with improved cohesion at 100 μm, reflecting moderate enhancement in gel formation and pore filling as higher GW content supplied more Al and Si from feldspar and micas. On the other hand, GWS70 presented the dominant N-A-S-H gel and CaCO_3_ at 5 μm with minimum illustration of microcracks, evolved gel networks with improved pore filling, and presented the excessive SiO_2_ particles reflecting the highest geopolymerization and enhanced structural integrity. This is because alkali activators derived from the dissolution of Si and Al from granite waste and reactive silica, leading to the formation of amorphous N-A-S-H gel that strengthened the matrix as well as helped the CaCO_3_ to act as a nucleating agent to support hybrid gel phases [43]. Furthermore, the densification of GWS70 resulted in higher mechanical strength in comparison with other mixes due to better adhesion and strong inter particles phases and structural bonding. This enhanced phase was also noticed in a previous study—that increasing the content of granite waste, partial amorphization of crystalline phases, especially quartz and feldspar, enhances gel particles adhesion and reduces the porosity up to 20% through better void occupancy by reaction products and unreacted fillers [44]. Similar enhancement of structural formation, improved crack resistance, and the highest mechanical strength were observed in this study due to strong N-A-S-H gel formation and less agglomeration effect, especially in GWS70 [45].

In comparison, GWS50 displayed a more porous, cracked, and weakly bonded structure due to limited reactivity from silica dominance, whereas GWS60 achieved moderate densification and pore refinement through increased GW content. The most prominent and strong structural formation was observed in GWS70 with the highest matrix cohesion and strong gel formation despite the microcracks due to shrinkage, making it the most ideal mix for geopolymerization efficiency and high mechanical strength.

### 4.4. Compressive Strength

**Figure 7:**
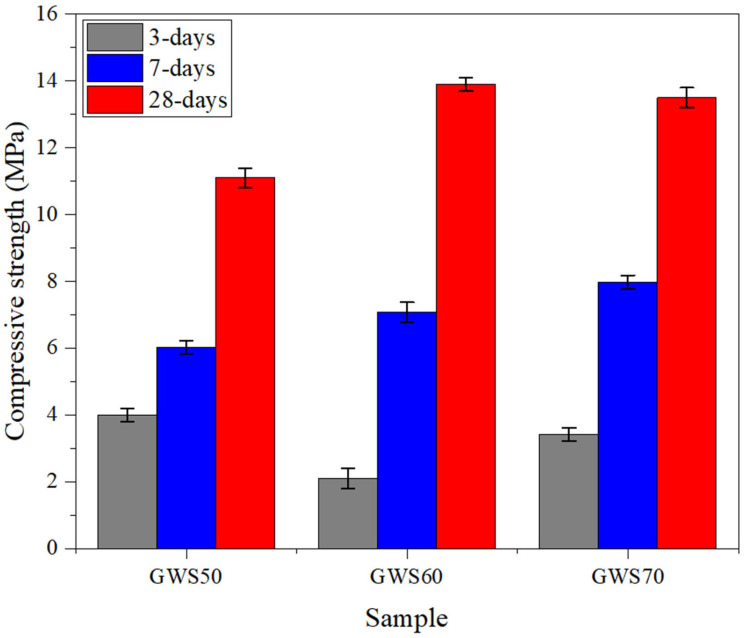
Mechanical strength of different mixes of GWS under constant loading.

The compressive strength results of alkali-activated granite waste with reactive silica (GWS) in different ratios revealed a distinct optimum composition at the intermediate granite waste replacement level, accompanied by different early-age and long-term strength development characteristics as revealed in Figure 7. At 3 days of curing period, GWS50 exhibited the highest strength of 4 MPa, when compared to GWS60 (3.5 MPa) and GWS70 (2 MPa), indicating that higher silica content initially promoted faster reaction because amorphous silica dissolved rapidly in alkaline activators with releasing abundant silicate monomers to form an early age gel framework, resulting in higher early strength [35]. However, at 7 days and 28 days curing, a reverse phenomenon was observed, as GW60 attained the highest compressive strength of 14.5 MPa compared to GWS50 and GWS70 at 28 days, reflecting that dominant role of aluminum availability from granite waste in formation of strong cross-linked 3-dimensional N-A-S-H gel while the optimum ratio of Si/Al resulted in the most cohesive and dense microstructure as seen in previous results [27].

GWS60 provided the ideal balanced and sufficient quantity of silica along with an adequate quantity of aluminum from granite waste to achieve sustained geopolymerization. Meanwhile, GWS50 showed a deficiency in aluminum due to a smaller quantity of granite waste with silica-rich gel formation, which led to longitudinal microcracking and poor bonding formation in comparison with other sample mixes, resulting in lower compressive strength overall. The observed performance at 60 wt% of granite waste illustrated consistent performance with previous studies, while other replacement ratios of granite waste, especially GWS70, delivered stable maximum mechanical strength at 28 days due to optimized reactivity for strong permanent gel formation and reduced porosity [46]. Overall, the compressive strength of GWS60 at 28 days illustrated a 32% increase in comparison with the strengths of GWS50 and GWS70. Thus, from the results, it was concluded that higher silica accelerated the initial reactivity for high early strength achievement, whereas GWS60 revealed stable resistance and optimal performance against continuous loading with strong mechanical properties.

## 5. Conclusions

This study evaluated the usage of granite waste with reactive silica in different proportions. The study introduced the new alkali-activated pastes as cementitious materials for applicability in construction applications. Based on the experimental results, the following conclusion can be drawn from this study:

i. ICP-MS analysis confirmed the highly complementary composition of calcined granite waste and reactive silica with balanced Si/Al ratios fulfilling the effective geopolymerization requirements.
ii. XRD patterns of GWS mixes revealed that increasing granite waste content from 50 wt% to wt70% enhanced the degree of geopolymerization and alkali gel formation by amplifying the feldspar peaks (orthoclase and albite), resulting in improved chemical stability. However, the higher GW content compromised the mechanical stability and microstructural integrity in GWS70 due to diminished quartz and α-cristobalite phases, leading to weaker bonding. Optimizing GW content from 50 to 60 wt% with reactive silica offered a balanced, sustainable approach for a high-performance alkali-activated mix.
iii. Functional groups analysis demonstrated that increasing calcined granite waste content significantly weakened Si-O-Si bands while intensifying O-H and H-O-H vibrations, confirming the effect of alkali activators on geopolymerization and shifts in functional groups formation. These alterations transformed the initial silica-rich (GWS50) into highly geopolymerized matrices in GWS60 and GWS70 by increasing chemical reactivity, leading to strong gel formation; however, increased GW content affected the functional performances of GWS mixes noticed from sharp peaks in GWS70.
iv. Compressive strength results showed that GWS60 delivered the highest compressive strength of 14.1 MPa, a 32% increase in strength over GWS50 and GWS70 by achieving the optimal balance of Si/Al ratio promoted the dense N-A-S-H gel formation. GWS50 exhibited faster early strength gain due to excessive reactive silica, while GWS70 exhibited lower strength as compared to GWS60 due to Al/Ca supply effecting the mechanical strength by revealing microcracks on the sample surfaces.

The present study demonstrated that the incorporation of calcined granite waste (GW) as a primary aluminosilicate precursor with reactive silica enables the successful development of a sustainable alkali-activated paste with excellent mechanical performance and reduced environmental impacts compared to traditional Portland cement. Among the investigated mixtures, GWS60 emerged as the optimum formulation by achieving the highest 28-day compressive strength of 14.1 MPa. These findings validated the combination of granite waste with highly reactive silica as a scientifically grounded and highly effective strategy for valorizing granite processing residues. The optimized GW-based alkali-activated mixes not only eliminate Portland cement entirely, but also substantially reduce energy consumption and CO_2_ emissions with concrete production, while delivering mechanical properties suitable for structural applications. The work thus establishes a robust, environmentally advantageous pathway for large-scale utilization of granite waste in high-performance, low-carbon construction materials.

## Figures and Tables

**Table 1:** Mix design ratio.

		Alkali-Activators (g)	
Group	Mixture Components	NaOH	Na_2_SiO_3_
GS50	GW50% + RSP50%	120	70
GS60	GW60% + RSP40%	120	70
GS70	GW70% + RSP30%	120	70

## Data Availability

The original contributions presented in this study are included in the article. Further inquiries can be directed to the corresponding author.
